# Assembly of the mitochondrial genome of the hydrothermal vent crab *Segonzacia mesatlantica* and detection of potential nuclear pseudogenes

**DOI:** 10.1080/23802359.2017.1318674

**Published:** 2017-05-19

**Authors:** Perrine Mandon, Laetitia Aznar-Cormano, Stéphane Hourdez, Sarah Samadi

**Affiliations:** a Muséum National d’Histoire Naturelle, Sorbonne Universités, Institut de Systématique, Evolution, Biodiversité, (ISYEB – UMR 7205 – CNRS, MNHN, UPMC, EPHE), Paris, France;; b Sorbonne Universités, UPMC Univ Paris 6, Station Biologique de Roscoff, Roscoff, France;; c CNRS, UMR 7144, Adaptation et Diversité en Milieu Marin, Station Biologique de Roscoff, Roscoff, France

**Keywords:** Mitochondrial genome, *Segonzacia mesatlantica*, Bythograeidae, Pseudogenes, Next-generation sequencing, Mid-Atlantic ridge vents

## Abstract

We assembled the mitogenome of the Bythograeid crab *Segonzacia mesatlantica*, using long-range amplification of the mitochondrial genome. The mitogenome is 15,521 base pair long (33.8% A, 21.7% C, 10.5% G, 34% T) with 13 protein-coding genes, 2 ribosomal RNA genes, 22 transfer RNAs, and a 624 bp AT-rich region. The gene arrangement is similar to other Brachyuran species. A whole genome shotgun sequencing approach revealed the presence of mitochondrial pseudogenes in the nuclear genome. This fifth mitogenome for a species of Bythograeidae should help resolve the puzzling question of the evolutionary origin of a family limited to deep-sea hydrothermal vents.

## Introduction

With 6793 species in 93 families, the Brachyura is the most diverse infra-order of Decapoda (Ng et al. 2008), but mitogenomes are available only for 51 species covering 22 families. We provide a sequence for the hydrothermal vent Bythograeidae crab *Segonzacia mesatlantica* Williams, 1988. *Segonzacia* is a monospecific genus and *S. mesatlantica* is the only crab found at Mid Atlantic Ridge vent sites (Desbruyères et al. 2006). Specimens were collected from Snake Pit (23°22′6.32″N; 44°57′ 11.66″W) and TAG (26°08′14.07″N; 44°49′33.75″W) during the BICOSE2014 cruise. DNA was extracted from the cheliped of two specimens (MNHN-IU-2013-15617 & MNHN-IU-2013-15603).

We aligned the four available Bythograeidae mitogenomes (Yang et al. 2010; Yang et al. 2013; Kim et al. 2013; Kim et al. 2015) and used Genious v.9.1.7 (Kearse et al. 2012) to define nine primers in addition to three universal primers LCOI, HCOI (Folmer et al. 1994), and 12S-R (Simon et al. 1994). These primers were used to amplify the whole mitogenome using a long-range amplification protocol. The products were sequenced using an Ion Torrent personal machine (PGM) (Life Technologies, Carlsbad, CA) with the Ion PGM™Hi-Q™ Sequencing Kit (Hinsinger et al. 2015). Reads were assembled by alignment with other Bythograeidae species.

The assembled mitogenome (KY541839) is 15,521-bp long. The base contents are similar to that of other Bythograeidae mitogenomes (33.8% A, 21.7% C, 10.5% G, and 34% T). The annotation and boundaries of protein-coding genes (PCGs), determined with Geneious and checked with MITO webserver (Bernt et al. 2013b), were refined by manually checking for consistent reading frame (ORF). The genome contains 13 PCGs, 2 ribosomal RNA genes, 22 transfer RNAs, and a putative AT-rich control region of 624 bp. The gene order is similar to most brachyurans, including the tRNA gene arrangement linking Crustaceans and insects (Boore et al. 1998). Two tRNA-Leu (TAA and TAG anticodons) and two tRNA-Ser (TCT and TGA) are found. The coding genes have ATC, ATG, ATT or GTG as start codons and TAA, TAG or T- (extended to TAA during posttranscriptional polyadenylation) as stop codons. Compared to other Bythograeidae, we found 20 instead of 5–9, non-coding intergenic nucleotides between tRNA-Leu (UAA) and COX2. Compared to other Bythograeidae, the COX2 gene is shorter, because of an early stop codon.

Libraries for a whole genome shotgun sequencing approach were generated using NEBNext^®^ Fast DNA Fragmentation & Library Prep Set for Ion Torrent™ (E6285L, New England Biolabs), loaded onto Ion 316V2 chips and also sequenced on a Ion PGM Sequencing platform (Hinsinger et al. 2015) and yielded 347,961 reads with an average of 160 pb. Only 397 reads were successfully mapped on the newly assembled mitogenome (allowing 20% maximum mismatch per read). Each read was manually checked. Reads diverging by only a few nucleotides (sequencing errors or intra-specific polymorphism) were considered as mitochondrial reads. Reads for which the ORF gave misplaced stop codons or different amino acid pattern were considered as potential nuclear mitochondrial pseudogenes. Such potential pseudogenes were found for most of the genes. COI pseudogenes have already been reported for other Bythograeidae crabs (Kim et al. 2013).

**Figure 1.**
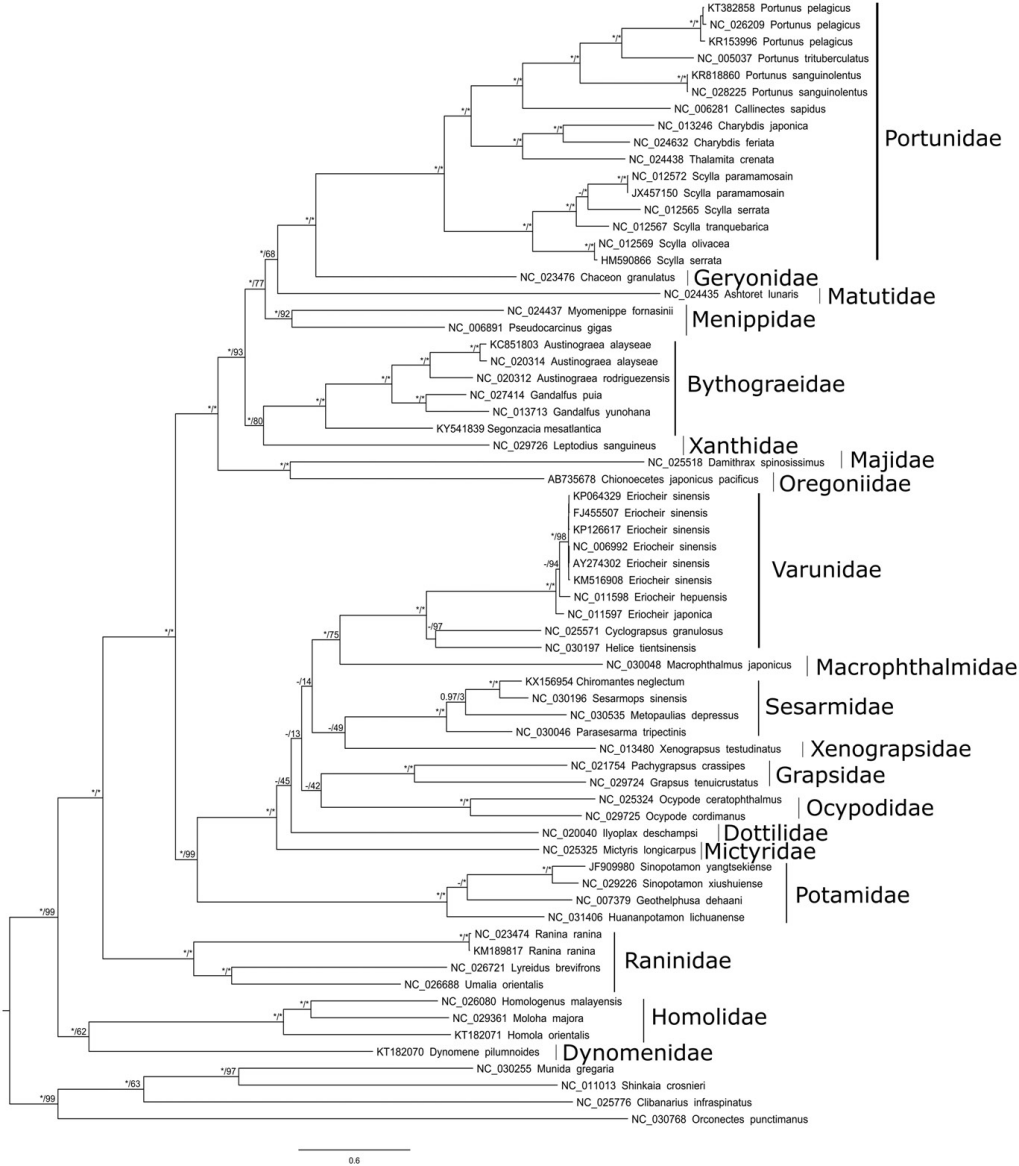
RaxML topology of Brachyura phylogenetic relationships based on the alignment of the concatenated 13 mitochondrial coding genes for 63 Brachyuran and 4 non-Brachyuran decapods. The model GTR + G + I was used in both maximum likelihood and Bayesian analysis. 500 rapid bootstrap resampling were set for RaxML analysis. Convergence of the 2 run Bayesian analysis was verified by the ESS values >200 with Tracer V.1.6. Node values correspond to Bayesian posterior probabilities/bootstrap. Asterisks indicate nodes with complete support in the considered analysis. A hyphen denotes a node not recovered in the considered analysis. All non-redundant available complete mitogenomes were selected and a primary RaxML analysis with a concatenated dataset was performed. The Leucosiidae *Pyrhila pisum* (NC_030047) generates a long branch and was thus excluded. Each gene was then separately analysed to detect potential long-branch artefacts. This analysis conducted to exclude the Cytb and ND6 genes of *Metopaulias depressus* (NC_030535). The dataset was completed with *Sinopotamon yangtsekiense* (JF909980) for which the sequences of ND1 and ND2 are lacking.

A preliminary phylogenetic tree is provided based on the alignment of the 62 available and non-redundant brachyuran mitogenomes (Figure 1). Based on this very incomplete taxonomic sampling, the monophyly of Bythograeidae is well supported but relationships with other families are not resolved.
